# The femoral trochlear anterior line is a better alternative intra-operative reference compared to femoral anterior tangent line for femoral rotation in both genders in total knee arthroplasty

**DOI:** 10.1186/s40634-020-00259-1

**Published:** 2020-06-08

**Authors:** Ji-Hoon Nam, Yong-Gon Koh, Paul Shinil Kim, Kiwon Kang, Kyoung-Tak Kang

**Affiliations:** 1 Department of Mechanical Engineering, Yonsei University, 50 Yonsei-ro, Seodaemun-gu, Seoul, 03722 Republic of Korea; 2 Joint Reconstruction Center, Department of Orthopaedic Surgery, Yonsei Sarang Hospital, 10 Hyoryeong-ro, Seocho-gu, Seoul, 06698 Republic of Korea; 3 Department of Orthopaedic Surgery, The bone hospital, 67, Dongjak-daero, Dongjak-gu, Seoul, Republic of Korea; 4 Orthopaedic Clinic, Gaja Yonsei Hospital, A-304,7, Janggogae-ro 337 beon-gil, Seo-gu, Incheon, Republic of Korea

**Keywords:** Korean patients; femoral rotation, Morphometry, Total knee replacement

## Abstract

**Purpose:**

To determine the most reliable reference axis for the femoral component rotation in TKA patients by comparing the trochlear anterior line (TAL) and the femoral anterior tangent line (FAT). To evaluate the variability of each anatomic parameter in a Korean population.

**Methods:**

Magnetic resonance images (MRIs) were taken for 500 patients (400 females and 100 males) with knee joint osteoarthritis who had Kellgren and Lawrence grade 3 and 4 prior to TKA in our institution between February 2016 and September 2017. It was investigated that whether significant differences in variance and gender exist for TAL and FAT.

**Results:**

TAL and the FAT were internally rotated by 5.1° ± 3.1° and 6.8° ± 6.1°, respectively, about the Transepicondylar axis (TEA). Although no gender-related differences were found for the TAL, they were found for the FAT. The variance of the TAL with respect to the TEA was significantly smaller compared with that for the FAT and thus exhibited a more consistent distribution. In addition, such a trend was found for both genders.

**Conclusions:**

The results show that the TAL is a favorable index for appropriate rotational alignment of the femoral component in TKA.

## Introduction

Femoral rotational malalignment is a major cause of patellofemoral complications, such as anterior knee pain and poor patellar tracking [1, 4, 12]. Various rotational axes have been proposed to perform appropriate rotational alignment of the femoral component, including the posterior condylar axis (PCA), the antero-posterior (AP) axis (Whiteside’s line), and the transepicondylar axis (TEA) [23]. Anatomical and biomechanical studies have shown that the TEA approximates the flexion–extension axis of the knee and that it is the optimal landmark for determining the femoral component rotation [13, 16]. However, it is not practical to use this axis because it is difficult to precisely locate the medial epicondyle and the sulcus during surgery [20]. In addition, it is challenging to apply such traditional references during surgery because arthritic changes, such as deformities, bony defects, and osteophytes, not only make it difficult to identify these references, but also distort them [3]. Recently, researchers have proposed two reference axes in the anterior femur as alternatives when conventional reference axes are ill-defined or distorted. The trochlear anterior line (TAL) is the line connecting the anterior points of greatest protrusion of the femoral medial and the lateral condyles [9, 17, 25], whereas the femoral anterior tangent (FAT) line is a line parallel to the anterior surface proximal to the point where the femoral trochlea ends [21, 23]. Both reference axes can be used for determining rotational alignment in the femoral component and have been regarded as useful indexes [21, 23, 25]. Watanabe et al. showed that the anterior surface of the femur immediately proximal to the trochlea and its FAT line could be used as a good index for the rotational alignment of the femoral component [23]. They analyzed preoperative computed tomography (CT) images of 150 knees with osteoarthritis and reported that the femoral anterior tangent line was consistently determined to be internally rotated approximately 12° to the TEA [23]. In addition, they recently developed a jig to measure the FAT line during surgery and examined the relation between preoperative and intraoperative measurement values [24]. They demonstrated that the FAT line is a useful index for appropriate rotational alignment of the femoral component both before and during TKA [24]. In contrast, Ji et al. reported that the variance of the TAL with respect to the TEA was significantly smaller than that of the FAT with respect to the TEA using CT images from 75 female Korean patients [8]. They demonstrated that the TAL is more reliable as an alternative reference for femoral rotation than the FAT [8]. However, the aforementioned study not only suffered from a small sample size, but gender differences were not analyzed.

The purpose of this study was to evaluate the relation between the TAL and the FAT and to determine which reference axis is more reliable by comparing the variances between the two lines.

## Material and methods

This study was retrospective review of the MRI of case series and approved by the ethics committee of the review board (IRB No. 18-DR-03). Five hundred twenty-four consecutive Korean patients who had Kellgren and Lawrence grade 3 and 4 and received TKA at our institution from February 2016 and September 2017 for osteoarthritis of the knee were included. Patients with any history of previous surgery or trauma on the knee joint were excluded. Thus, morphological measurements were performed in 500 patients with knee joint osteoarthritis; 100 males and 400 females. The mean patient age was 69.4 ± 7.2 years for male and 70.0 ± 6.2 years for female. The mean body mass indexes of the female and male patients were 24.3 ± 3.2 kg/m^2^ and 23.2 ± 3.4 kg/m^2^ (Table 1), respectively.

**Table 1:** Comparison of the age and BMI between Korean males and females

Parameter	Whole patients (*n* = 500)	Female (*n* = 400)	Male (*n* = 100)	*p*-value
	Mean ± SD	Mean ± SD	Mean ± SD	
Age	69.9 ± 6.4	70.0 ± 6.2	69.4 ± 7.2	n.s
BMI (kg/m^2^)	24.2 ± 3.3	24.3 ± 3.2	23.2 ± 3.4	n.s

*n.s* non-significant

MRIs were acquired using a 1.5-T MRI scanner (Achieva 1.5 T; Philips Healthcare, Best, Netherlands). They were obtained using a high-resolution slice with a thickness of 1 mm in the sagittal plane for the tibiofemoral knee joint and a slice with a thickness of 5 mm in the axial plane for the hip and ankle joints. Under non-fat saturation conditions, the MRIs consisted of axial proton density sequences. A high-resolution setting was used for the spectral pre-saturation inversion recovery sequence (echo time, 25.0 ms; repetition time, 3590.8 ms; acquisition matrix, 512 × 512 pixels; number of excitations (NEX), 2.0; field of view, 140 × 140 mm). This approach, which is used in patient-specific instruments, allowed us to effectively develop 3D reconstructed models [18]. The MRIs were imported into a modeling software (Mimics version 17.0; Materialise, Leuven, Belgium) and segmented to construct 3D bony and cartilage models of the femur. A 3D reconstruction reproducibility analysis was performed in a manner similar to our previous study [11].

**Fig. 1:**
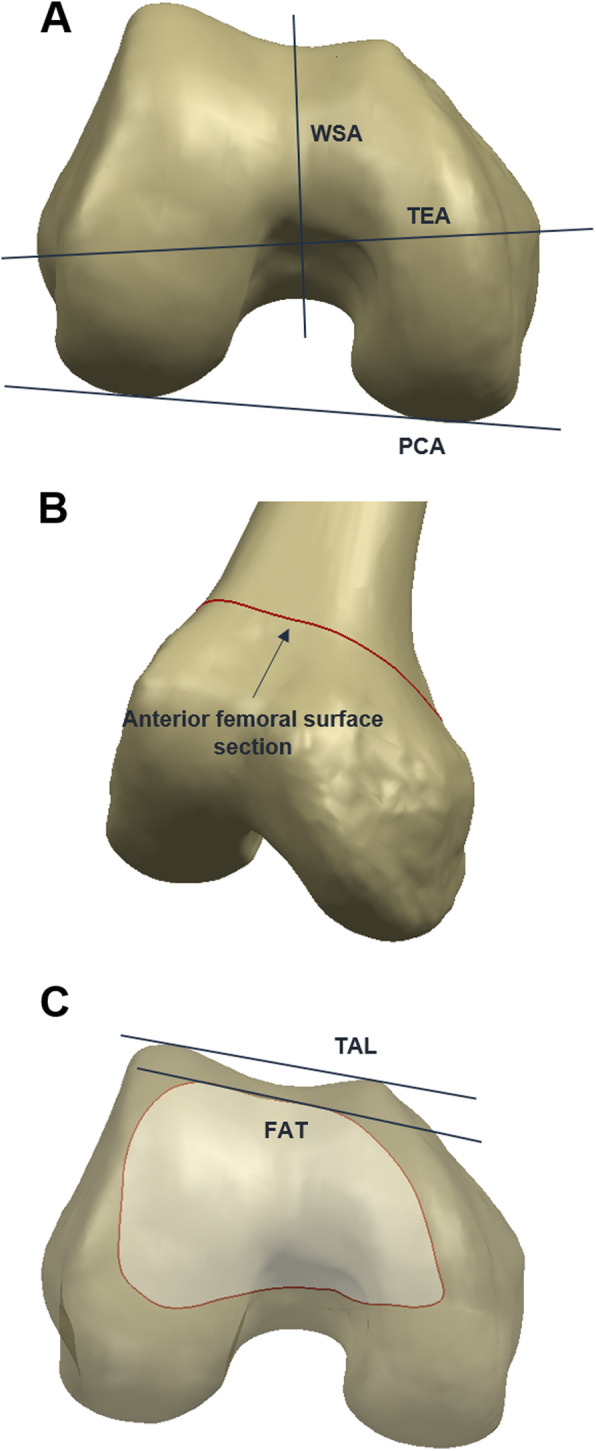
Schematic representation of (**a**) TEA, PCA, WSA, (**b**) Anterior femoral surface section which indicate the immediate proximal section line, and (**c**) TAL, FAT

On the reconstructed femur models, anatomical landmarks were selected to create anatomical axes. Three anatomical axes were created for performing measurements (Fig. 1). The mechanical axis was created using the intercondylar notch point and the hip center. The TEA axis was created using the medial and lateral epicondyles. The TAL was created using the most anterior point of the medial and lateral femur anterior condyles [8]. The FAT line was identified in the following fashion [23]. First, the immediate proximal section line was created. Then, the line tangent of the anterior side of the section line was created. The angles between TAL/TEA and FAT/TEA were measured. All measurements were performed by a single experienced observer. To assess intra- and interobserver variability, 100 3D MRI scans from 50 female and 50 male patients were re-measured more than 1 week after the initial measurement by the same observer and by a second observer.

### Statistical analysis

The statistics of the data were analyzed using SPSS (version 18.0; SPSS, Chicago, IL, USA), and Student’s *t* tests were carried out to evaluate the significance of the differences between genders for the 400 female and 100 male patients. The normality test was conducted to assess the normality. The variances between the angles of the TAL and FAT lines with respect to the TEA were compared via F-tests. A *p*-value < 0.05 was used to define statistical significance. The post hoc power analysis was conducted using G*power. Difference between male and female groups in FAT was used as statistical parameters. The alpha value was 0.05 and the output parameter was 0.98.

## Results

The intraobserver error (0.89) and interobserver error (0.92) were calculated using the intra-class correlation method. All demographic data and measured angles were found to have a normal distribution, and all statistical values were evaluated through averages and standard deviations. We found no significant differences in the demographic factors, including age and the body mass index, between the genders (Table 1). There were no gender differences for the TAL with respect to the TEA (Table 2).

**Table 2:** Comparison of anthropometric measurements between Korean males and females

Parameter	Whole patients (*n* = 500)	Female (*n* = 400)	Male (*n* = 100)	*p*-value
	Mean ± SD (range)	Mean ± SD (range)	Mean ± SD (range)	
TAL/TEA(°)	5.1 ± 3.1 (−7.5,18.2)	5.0 ± 3.2 (− 7.5,18.2)	5.6 ± 2.6 (0.0,12.4)	n.s
FAT/TEA(°)	6.8 ± 6.1 (− 14.0,21.6)	6.3 ± 6.3 (− 14.0,21.6)	8.8 ± 5.3 (− 14.0,21.6)	< 0.05

*n.s* non-significant

As for the FAT, gender differences were found (*P* < 0.05).

**Fig. 2:**
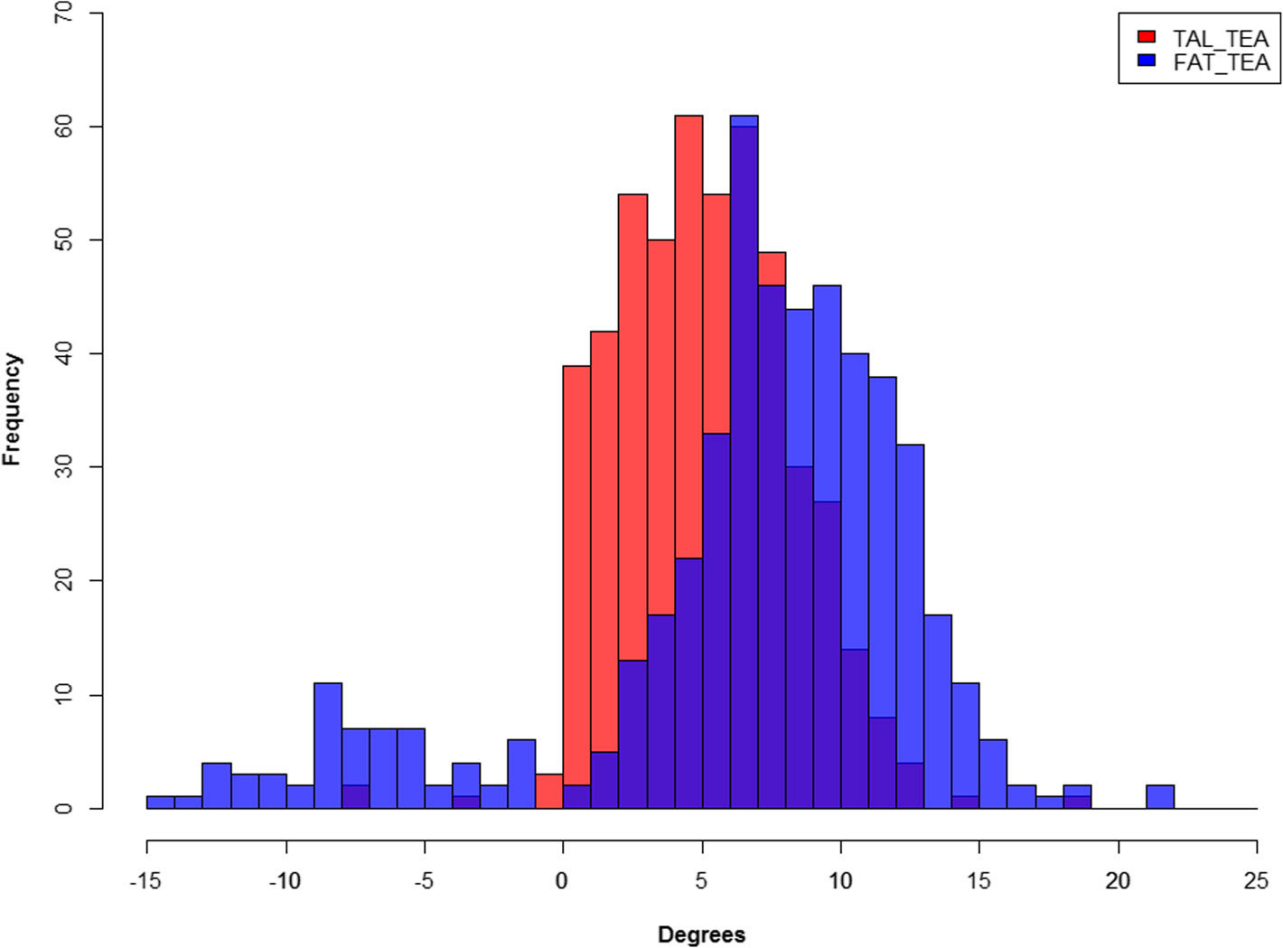
Distribution of the TAL/FAT and FAT/TEA angles

Figure 2 shows that the relationship between the FAT and the TEA exhibited higher variability compared with that of the TAL and the TEA. After comparing the variances between the TAL with respect to the TEA and the FAT with respect to the TEA, the *p* value was found to be 0.05, which means that the variance of the TAL with respect to the TEA was significantly smaller than that of the FAT with respect to the TEA. This trend was found in both male and female patients.

## Discussion

The most important finding in this study was that variance in the TAL was smaller than that of the FAT, which means that it is more appropriate to use the TAL rather than the FAT as an alternate femoral rotational axis. In addition, no gender differences were found in the TAL, meaning that it could be used as a femoral rotational axis regardless of gender.

Owing to the advances in the development of the TKA instruments, the frequency of placements of femoral components with an inappropriate rotation has decreased [14]. However, the rotation provided by such instruments may not be ideal, and thus surgeons should carefully determine relevant anatomical parameters for correct implant positioning [14]. Despite the availability of radiographic images for correct implant positioning, unsatisfactory clinical outcomes occur for some patients. In consideration of these unfavorable and unexpected situations, it has been proposed that such outcomes could be ascribed to constitutional alterations specific to a certain race or gender [14]. This should be verified by analyzing anatomical differences between populations [4, 5].

In this context, some studies have shown rotational differences between female and male patients. Previous studies have shown that the TEA and the AP axis are reliable axes for determining femoral rotation and that the TEA was the most reproducible landmark leading to the best balance [18], whereas the posterior femoral condyles were less reproducible for determining femoral rotation.

Various bony landmarks may not always be identifiable on x-ray images or during surgery. The clinical TEA can be easily obtained in CT images, whereas the surgical TEA is not always readily determined. Previous studies have shown that the identification rate of the medial sulcus using CT images ranges from 20% to 74% [2, 22, 26], which may further decline in knees with severe osteoarthritis. The accuracy of TEA measurements may be hampered by soft tissue coverage or difficulty in accessing the lateral condyle as well as by other surgical factors [7, 23]. The FAT lines obtained in a previous study exhibited relatively invariable internal rotation with respect to the clinical TEA [23]. Twelve degrees of external rotation with respect to the FAT line during TKA would properly approximate the femoral rotational alignment to the TEA [23]. In addition, the FAT line, as well as the AP axis, were not affected by varus–valgus deformities in osteoarthritic knees [23]. However, their study did not include the angle between the surgical and clinical TEAs. They considered the angle between the PCA and the surgical TEA to be approximately 3°, whereas the angle between the FAT and the TEA should be 9.2° ± 3.6° [23]. Nevertheless, the FAT internal rotation angle determined in this study is smaller than that indicated in previous studies [8, 23]. However, these researchers have recently measured the FAT intraoperatively, and they found that it was internally rotated by 7.3° ± 4.0° with respect to the TEA [24]. This is very close to the value we obtained in this study, which was 6.8° ± 6.1°. It should be nonetheless noted that anthropometric characteristics are related to genetic, environmental, and sociocultural conditions and to lifestyle, health, and functional status [6]. These variations make it challenging to provide a standard interpretation of their respective values [6].

Talbot and Bartlett showed that the TAL has a close correlation to the TEA and, for the first time, established an alternative to the direct visualization of the TEA [21]. The TAL was internally rotated by 7.3° ± 1.8° with respect to the TEA in healthy knee joints, which is approximately 1° more internally rotated than in our study [25]. However, in patients with arthritis, this value has been reported to be 5.6° ± 2.3° [17] and 6.1° ± 2.5° [8], which is comparable to our results.

After comparing the variances between the FAT and the TAL, the latter exhibited a more consistent distribution. The anterior protrusion of the lateral condyle is more anterior and larger than that of the medial condyle, and thus the distribution of the TAL with respect to the TEA is uniform. Such a trend was also found in a previous study [8].

A cadaveric study showed that the median surface of the cortical bone may be depressed or protruded, leading to negative values of the FAT with respect to the TEA [19]. This could be the reason that the variance of the FAT with respect to the TEA tends to be larger than that of the TAL with respect to the TEA, implying that the TAL is a more reliable indicator of rotational alignment [8]. However, another recent study showed that the posterior cortical bone is a better landmark than the anterior cortical bone of the distal femur for indirectly determining the surgical TEA [15]. Data showed that the anterior cortical bone has a greater variance in relation to the TEA than the posterior cortical bone. This is attributable to the greater variability of the geometry of the distal femur in the anterior aspect compared with the posterior aspect [15].

Although we did not measure the posterior cortical bone line, the fact that the anterior cortical bone exhibited a greater variance in our study is in line with previous findings. Our results showed no gender differences in the TAL, and its variance was smaller than that of the FAT. Therefore, the TAL can be considered as a useful landmark.

There were three main limitations in this study. First, MRIs were used to construct 3D representations of the distal femur, Nevertheless, the MRIs allowed us to reconstruct soft tissues, such as the articular cartilage, and the inaccuracy in 3D reconstruction could be reduced using a protocol described in a previous study [10]. Secondly, the population under study lacked ethnic diversity, and the results might differ for other populations. Thirdly, this study does not provide postoperative clinical outcomes because we did not investigate patients who underwent TKA. Nevertheless, this study provides valuable information of alternative anatomical references for surgeons when the posterior condylar surface and the trochlear groove are worn and distorted.

## Conclusions

The TAL should be taken into account in all cases in which it is accessible without causing additional soft tissue trauma. Our results showed that the TAL is a favorable index for appropriate rotational alignment of the femoral component in TKA because TAL has smaller variance than FAT. This information is useful for performing femoral rotation alignment when conventional reference axes, such as the PCA or the AP axis, are not available.

## Data Availability

Not applicable.

## Abbreviations

TKA: Total Knee Arthroplasty
TAL: Trochlear Anterior Line
FAT: Femoral Anterior Tangent Line
MRIs: Magnetic Resonance Images
TEA: Transepicondylar Axis
PCA: Posterior Condylar Axis
AP: Antero-Posterior
